# Supplementary material for: Abundant, diverse, unknown: Extreme species richness and turnover despite drastic undersampling in two closely placed tropical Malaise traps
Source: PLoS One. 2023 Aug 16;18(8):e0290173. doi: 10.1371/journal.pone.0290173 (PMC10431641; doi:10.1371/journal.pone.0290173)
Supplement: S1 Table — (PDF) [file pone.0290173.s002.pdf]

S1 Table. Statistical analysis of the community compositions.

		PERMANOVA				Permutest
Variable	Df	SS	R^2	F	Pr>(F)	P
Malaise trap	1	1.5840	0.22148	5.6899	0.001	0.689
Residual	20	5.5677	0.77852			
Total	21	7.1517	1			
