# Supplementary material for: Abundant, diverse, unknown: Extreme species richness and turnover despite drastic undersampling in two closely placed tropical Malaise traps
Source: PLoS One. 2023 Aug 16;18(8):e0290173. doi: 10.1371/journal.pone.0290173 (PMC10431641; doi:10.1371/journal.pone.0290173)
Supplement: S1 Fig — NMDS plot of the insect community compositions within each collection sample. Ellipses are 95% confidence intervals of centroids for each Malaise trap. (PDF) [file pone.0290173.s001.pdf]

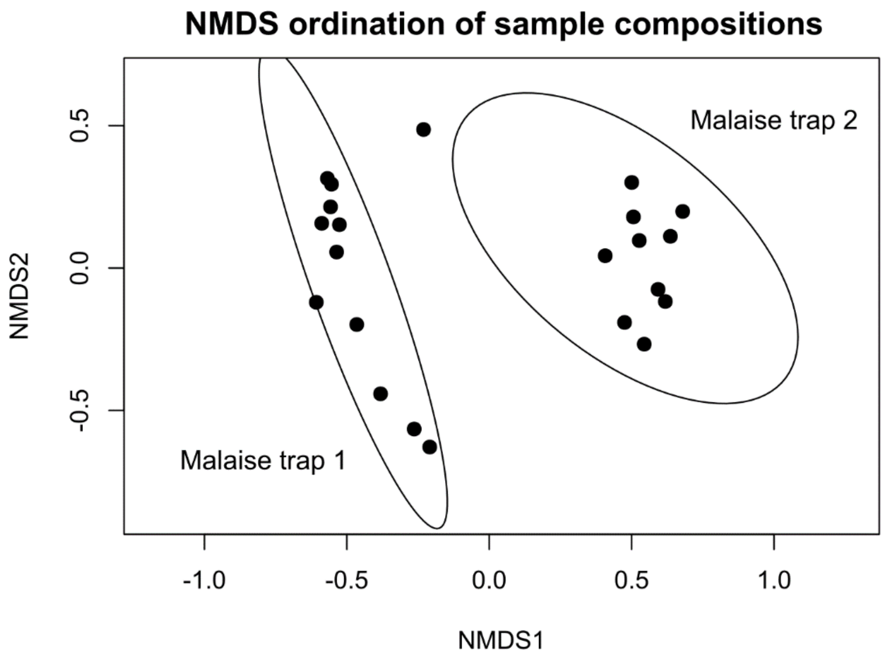

S1 Fig. Non-metric dimensional scaling (NMDS) of the community compositions. NMDS plot of the insect community compositions within each collection sample. Ellipses are 95% confidence intervals of centroids for each Malaise trap.
