# Supplementary material for: Abundant, diverse, unknown: Extreme species richness and turnover despite drastic undersampling in two closely placed tropical Malaise traps
Source: PLoS One. 2023 Aug 16;18(8):e0290173. doi: 10.1371/journal.pone.0290173 (PMC10431641; doi:10.1371/journal.pone.0290173)
Supplement: Attachment — Submitted filename: Response to Reviewers.docx [file pone.0290173.s003.docx]

Reviewer #1: Review of ‘Abundant, diverse, unknown: Extreme 1 species richness and turnover despite drastic undersampling in two closely placed tropical Malaise traps’

I can find very little to criticise in this manuscript. I suppose that none of the results are particularly surprising, as the authors acknowledge, but the empirical demonstration of massive local heterogeneity and very high species richness are very useful, and the methods will be very useful for the development of further studies at different scales. I would hope that the availability of large numbers of barcodes for ‘dark taxa’ would also promote further taxonomic work.

For those samples for which you filled 15 plates, rather than all specimens, what was the protocol for choosing individuals to sequence? Or were specimens chosen randomly until the plates were full?

Author response:

Since the main aim was to maximize the number of BINs from the Indonesian Malaise traps, a selective strategy was used to narrow down the number of specimens especially for the samples with only 15 plates processed. Individuals chosen for sequencing were selected to capture as much diversity as possible based on size and morphospecies. Two sizes of sieves were used to subsample from three different size classes (no sieve, 8mm sieve, and 2mm sieve). As most of the diversity was likely hidden in the smaller organisms (particularly the abundant insect orders: Hymenoptera, Coleoptera, and Diptera), more specimens were chosen from the smallest size class. Additionally, more Hymenoptera and Coleoptera were selected as opposed to Diptera because Diptera are often so abundant in Malaise trap samples that there is a higher risk of oversampling the same species.

We have added this information to the manuscript, see lines 96-105.

What happens to the specimens? Are they available for taxonomic work?

Author response:

All barcoded specimens are currently stored at the Center for Biodiversity Genomics (CBG) natural history archive (collection code BIOUG) at the University of Guelph, Canada. However, this collection, as well as the rest of the unprocessed material, will eventually be repatriated to Museum Zoological Bogoriense in Cibinong, Indonesia. We have also added this to the manuscript, see lines 114-117.

I couldn’t find the datasets which are deposited on BOLD. Are they publicly available yet? I was hoping to scan through the areas I know best (Ichneumonoidea) and sense check the results.

Author response:

In the meantime, the DOIs went public and are now accessible under the following links: dx.doi.org/10.5883/DS-GMTINDO1 and dx.doi.org/10.5883/DS-GMTINDO2.

Lines 46-47: I don’t think ‘dark taxa’ need be defined by a complete absence of taxonomic expertise, but clearly the expertise is very limited and completely inadequate to the job.

Author response:

Thank you, we have made changes. See lines 46-47: …which is either in decline or very limited, the latter being the case in the so-called dark taxa (6,7).

Change ‘an increase by…’, to ‘an increase of…’ (eg., line 205)

Author response:

Thank you, we have changed this, also throughout the manuscript.

Reviewer #2: The manuscript by Chimeno et al. compares the insect communities collected using two closely spaced Malaise traps in Sumatra. It presents a well sampled dataset of ~70K insects and compares these two sites in terms of alpha and beta diversity. The findings of high species diversity of insects along with the distinct communities in nearby sites are of interest for wider scientific community. However, I would suggest the following be considered:

The paper shows surprisingly distinct communities across the two sites despite their proximity. But the manuscript has a very limited description of the sites being compared (other than them being forest edge sites). It would be important to describe the two sites in the study in detail and the area around in general in the methods section of the manuscript.

Author response:

We have added more site information, see lines 79-88.

The manuscript furthermore characterizes the insect communities at family level but does not describe how the family level identifications were made for the insects. This should be properly documented in the methods. Similarly, standard barcode protocols of CCDB is cited at L93 but the link directs to several other links: It would be good if the major steps are described (target region, extractions, sequencing methods) given the linked protocols may change in future. Regarding data analysis, how were the sequences aligned?

Author response:

Thank you for addressing this. We have added the information to the Methods (see data analysis). All family-level identifications were conducted using the BIN taxonomy match tool on BOLD, and all sequences without information were excluded from these analyses. The BIN taxonomy match tool applies taxonomic identifications up to the lowest level of concordant taxonomy within all members of a BIN using the entire BOLD database as a reference library.

We have added more information to the laboratory methods. To summarize, the standard 658 base pair region of the cytochrome c oxidase subunit I (COI) gene was the target region. Steps included tissue lysis, DNA extraction, microplate consolidation into 384-well format (to reduce reagents and costs) before PCR amplification, cycle sequencing, and subsequent Sanger sequence analysis.

For your interest, more detailed sequencing steps are accessible from each sequence page on BOLD if you click on the LIMS icon at the bottom (see images).

While those familiar with the topic would not find the assumption that the specimens sequenced in this project (given they are from Southeast Asia and given the insect families they belong to) are from dark taxa ‘for which little taxonomic… information is available’ (L277) surprising, it is worth noting that the manuscript does not document this finding. It would be valuable to characterize how many of the OTUs sequenced in this project are (1) present in the barcode databases and (2) are identified to species level. This further links to the title of the paper abundant diversity and “unknown”.

Author response:

This is a very interesting point, thank you! We have added information to the manuscript, see lines 183 – 189. More than two-thirds (6,125) of all BINs were unique to BOLD, meaning that they were added for the first time with the upload of these sequences. Of the 58,769 specimens that were successfully sequenced, 961 obtained a species-level identification, providing coverage for 231 species.

We have also added (now) Figure 2 to the manuscript, depicting the frequency of rare and common BINs within the data, and also the proportion of these “unknown” groups.

A key issue relates to the discussion of the results. The authors find high diversity, distinct communities and that most species and specimens belong to a few families. However the discussion of the solution to these challenges is not developed; i.e. what are the recent methods that can be used to expedite the work for taxonomists and how can such large scale studies be conducted given that even 70K specimens are not sufficient for studying these sites? What is the proposal for future research, particularly in tropics?

Author response:

We have added more on this topic, and discuss several possibilities for expediting taxonomic work. See lines 370 -407.

This may be a preference, but I find that structurally the first section of the discussion “Employing DNA-based delimitation methods: working with 225 species proxies” is less interesting than several of the key findings described in the paper and it would be more interesting to highlight the key findings of the manuscript first.

Author response:

Thank you, we have rearranged all sections of the manuscript so that we first describe our key findings, then finish off with the comparison of the different clustering analyses.

Minor:
L152: The links are not working for me

Author response:

In the meantime, the DOIs went public and are now accessible under the following links: dx.doi.org/10.5883/DS-GMTINDO1 and dx.doi.org/10.5883/DS-GMTINDO2.

L244: -21% and -14% is confusing to me. 21% of 6283 (RESL) is 1319 and -21% would imply 4960 ASAP OTUs based on table 2, but doesn’t match 5185. Perhaps it should be +21% if the reference is ASAP OTUs. But overall, I would suggest not using % here given the clusters themselves are not necessarily the same. Same for L239.

Author response: This is a good point. We went ahead and removed the %.

L278-282: mentions eight families but lists only seven

Author response: Thanks!
