# Supplementary material for: Abundant, diverse, unknown: Extreme species richness and turnover despite drastic undersampling in two closely placed tropical Malaise traps
Source: PLoS One. 2023 Aug 16;18(8):e0290173. doi: 10.1371/journal.pone.0290173 (PMC10431641; doi:10.1371/journal.pone.0290173)
Supplement: Attachment — Submitted filename: Response to Reviewers.docx [file pone.0290173.s004.docx]

Reviewer #2: The authors have addressed my comments. Upon review, and looking through the links that are now available I have a few suggestions.

The numbers in BOLD datasets dx.doi.org/10.5883/DS-GMTINDO1 and dx.doi.org/10.5883/DS-GMTINDO2 correspond to 52239 and 24938 specimens (and strangely enough, sequences) which is slightly different from the numbers in L254 (52362 and 25135) and the actual number of sequences is far fewer. The downloaded sheets from figshare correspond to the values in the manuscript. Based on "BIN_data" from figshare, approximately 12,000 specimens have 0bp barcodes, but the number of sequences in the BOLD page seems to be too high, though upon download the sequence count gets closer, though specimen count is still off. I am not sure what is going on, it would be good if a field of sequence is added to one of the figshare spreadsheets for straightforward access to data for future users along with a flag of which sequences were used for final analysis.

Response: This is in fact a simple mistake – we realized that the BOLD support team has not transferred all specimens to the dataset that was published. We have contacted them and this will be changed!

The authors have stated that BOLD taxonomy is used for family level classification. Given these were not morphologically examined/verified, it would be good to mention in the discussion this limitation/potential errors propogating in sequence based identifications.

Response: This has been added to the discussion.

L361: Edit to "in temperate regions", and given temperate samples are not involved in this study, this should be cited, and the similarity in pattern has been quantified in <https://doi.org/10.1038/s41559-023-02066-0>

Response: Thank you, we have added this information also.
